# A VR-Based Trauma Nursing Education Program for Clinical Nurses: Integrating Jeffries’ Model and the 5E Learning Cycle

**DOI:** 10.3390/healthcare13192542

**Published:** 2025-10-09

**Authors:** Heeyeon Kim, Gyuli Baek, Eunju Lee

**Affiliations:** 1 College of Nursing, Keimyung University, Daegu 42601, Republic of Korea; hiri1986@naver.com; 2 College of Nursing, Yeungnam University College, Daegu 42168, Republic of Korea; koori2025@gmail.com

**Keywords:** clinical nurses, trauma nursing, virtual reality, Jeffries’ simulation model, 5E Learning Cycle

## Abstract

Background/Objectives: Nurses’ professional competencies are critical in trauma patient care, and educational programs that strengthen these competencies contribute to improved patient safety and higher-quality care. This study aimed to develop and evaluate the effectiveness of a virtual reality (VR)-based trauma nursing education program by applying Jeffries’ simulation model and the 5E Learning Cycle. Methods: A quasi-experimental, non-equivalent control group pretest–post-test design was employed. Participants were 34 nurses with more than one year of clinical experience, recruited from three university hospitals in Daegu, Korea, each with over 800 beds. Participants were allocated to either the experimental group (*n* = 17) or the control group (*n* = 17). The experimental group received the VR-based program, while the control group received standard training. Effectiveness was assessed using validated questionnaires measuring trauma-related knowledge, confidence in trauma care, and emergency nursing competency. Data were analyzed with repeated-measures ANOVA. Results: The experimental group demonstrated significant improvements in trauma-related knowledge and confidence in trauma care compared with the control group. Emergency nursing competency also increased significantly in both groups over time, but the degree of improvement did not differ between groups. Conclusions: The VR-based trauma nursing education program, designed using Jeffries’ simulation model and the 5E Learning Cycle, enhanced trauma-related knowledge and confidence among clinical nurses. Although no between-group difference was found for emergency nursing competency, the findings provide foundational evidence supporting the use of VR-based interventions to advance emergency and critical care nursing education.

## 1. Introduction

As societies advance and industrialization accelerates, the incidence of injuries—including road traffic collisions, falls, and workplace accidents—has steadily increased. Globally, injuries account for approximately 4.4 million deaths annually, representing nearly 8% of all deaths [1]. They also rank among the leading causes of disability-adjusted life years (DALYs), highlighting their profound impact not only on mortality but also on long-term disability [2]. Beyond the health burden, trauma imposes substantial economic costs due to treatment, rehabilitation, and lost productivity [3,4]. Effective emergency care systems and timely interventions are therefore critical for improving survival and reducing complications in trauma patients [5].

Within this context, nurses play a pivotal role by providing immediate life-saving interventions and comprehensive assessments to prevent secondary injuries and complications in trauma patients [6]. The concept of the “golden hour,” referring to the critical one-hour period after trauma during which prompt surgical and clinical interventions can significantly improve survival, further underscores the importance of rapid and precise care [7]. However, research indicates that many nurses lack sufficient knowledge and skills in trauma care [8,9]. While standardized training programs such as Advanced Trauma Life Support (ATLS) are recommended, their implementation is often limited by cost, time, and resource constraints [10]. These challenges highlight the need for innovative educational approaches that provide diverse trauma scenarios and flexible, repeatable learning opportunities. These challenges highlight the urgent need for innovative and accessible educational approaches that allow repeated practice across diverse trauma scenarios.

Simulation-based education offers a valuable pedagogical strategy to compensate for the limitations of clinical training. It enables repetitive practice in realistic environments using high-fidelity simulators or virtual reality, while also incorporating structured feedback from peers, instructors, and video review [11,12]. This method supports the safe acquisition of essential skills. Nevertheless, conventional simulation training is constrained by logistical barriers and often focuses on single emergencies (e.g., cardiac arrest) rather than complex trauma scenarios [13,14]. To address these limitations, trauma-focused simulation programs should incorporate diverse, clinically relevant cases and provide opportunities for repeated practice unconstrained by time and place.

Virtual reality (VR) simulation provides such flexibility, allowing learners to engage in three-dimensional, immersive environments accessible both in-person and online [11,15]. By replicating high-risk emergency settings, VR fosters learner engagement, clinical reasoning, and decision-making [16]. The integration of the 5E Learning Cycle—Engagement, Exploration, Explanation, Elaboration, and Evaluation—further enhances cognitive processing, promoting active knowledge construction and logical reasoning [17,18]. Nursing education research has demonstrated that the 5E model strengthens self-efficacy, decision-making, and critical thinking [19,20].

Although positive outcomes of VR simulation have been reported in areas such as respiratory care [20] and asthma management [21], most trauma-related VR studies have focused on specific technical procedures (e.g., physician training or intubation) rather than comprehensive nursing education [22,23]. This gap underscores the need for integrated VR-based programs specifically tailored to trauma nursing practice. Therefore, this study aimed to develop and evaluate a VR-based trauma nursing education program systematically designed using Jeffries’ simulation model [24] and the 5E Learning Cycle [17]. We hypothesized that this program would improve trauma-related knowledge, confidence in trauma care, and emergency nursing competency among clinical nurses.

## 2. Method

### 2.1. Study Design

This study employed a quasi-experimental design with a nonequivalent control group pretest–post-test structure.

### 2.2. Participants

The participants were nurses with at least one year of clinical experience, voluntarily consenting to participate in the study. They were recruited from three university hospitals, each with more than 800 beds, located in D Metropolitan City. The sample size was calculated using G*Power version 3.1, setting repeated-measures Analysis of Variance (ANOVA) for two groups with a medium effect size (f = 0.25), a power (1 − β) of 0.80, and a significance level (α) of 0.05. The minimum required sample size was 17 participants per group. Allowing for a 20% dropout rate, 21 participants were recruited for each group, totaling 42. Participants were allocated to the experimental or control group based on hospital affiliation to minimize contamination between groups. The experimental group received the VR-based trauma nursing education program, while the control group continued with standard in-service education without exposure to VR or simulation-based trauma training. Attrition was handled by applying an intention-to-treat principle during data collection; however, eight participants (four from each group) were excluded from the final analysis due to incomplete questionnaire responses and voluntary withdrawal. Thus, the final analysis included 34 participants (17 in each group).

### 2.3. Instruments

#### 2.3.1. Confidence in Performing Trauma Care

Confidence in trauma care was measured using an instrument originally developed by Kim [25] for professional resuscitation, which was modified and supplemented according to the Advanced Trauma Life Support (ATLS) guidelines and prior literature on trauma patient care. Content validity was established through two rounds of expert review by a panel comprising two trauma surgeons and two nursing scholars (all with doctoral degrees and an average of 12 years of teaching experience). Following their feedback, the items were refined, and the content validity index (CVI) was confirmed to be 1.0. Construct validity was additionally supported by expert consensus that the items appropriately reflected the cognitive and psychomotor domains of trauma care. The final instrument consisted of 10 items rated on a 10-point Likert scale (1 = “strongly disagree” to 10 = “strongly agree”), with higher scores indicating greater confidence in trauma care. In Kim’s study [25], Cronbach’s α was 0.92, while in the present study, it was 0.96. Test–retest reliability was examined in a subsample of 10 nurses over a two-week interval, yielding a correlation coefficient of r = 0.91, indicating excellent stability.

#### 2.3.2. Knowledge of Nursing for Patients with Trauma

Knowledge of trauma nursing was measured using a tool originally developed by Seo [26] for nursing students and revised for clinical nurses in this study. The original instrument comprised 20 items drawn from lecture notes on the Basic Trauma Care course: three items on trauma concepts, eleven on patient assessment, and six on trauma management. To refine item difficulty, a pilot test was conducted with six third-year emergency medical technician students, and revisions were made accordingly. Content validity was reviewed by two experts—one professor teaching Advanced Trauma Care in the Department of Emergency Medical Services and one professor in Critical Care Nursing Practice in the Department of Nursing. In addition to content validity, criterion-related validity was examined by correlating scores on this tool with clinical performance evaluation scores in a pilot group, showing a significant positive correlation (r = 0.68, *p* < 0.01). Each item was designed in multiple-choice format with five response options, including an additional “I do not know” option to control for guessing. Correct answers were scored as 1 point, and incorrect or “I do not know” responses as 0 points. Total scores ranged from 0 to 20, with higher scores reflecting greater knowledge. In the present study, the reliability (KR-20) was 0.84, indicating good internal consistency.

#### 2.3.3. Emergency Nursing Competency

Emergency nursing competency was measured using the instrument originally developed by Yook [27] and revised by Cho [8]. The final version consisted of 33 items across 12 subdomains, each rated on a five-point Likert scale (1 = “not at all” to 5 = “always”). Higher scores indicated greater levels of competency. In Cho’s study [8], the reliability was reported as Cronbach’s α = 0.96. In the present study, Cronbach’s α was 0.97, demonstrating excellent internal consistency. To further ensure reliability, inter-rater agreement was examined in scoring a subset of responses, yielding an intraclass correlation coefficient (ICC) of 0.89.

### 2.4. Research Procedures

#### 2.4.1. Theoretical Framework

**Figure 1:**
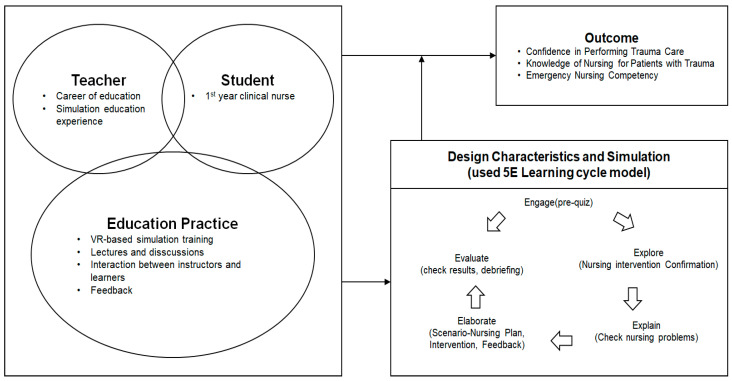
Theoretical framework of this study.

The trauma nursing education program was developed by applying the 5E Learning Cycle model [28], grounded in Jeffries’ simulation framework [24] (Figure 1). The instructor component, as specified by Jeffries’ model, was fulfilled by a nurse educator with more than five years of emergency nursing experience and expertise in simulation-based education. The learner component comprised clinical nurses with at least one year of professional experience. The program followed a blended learning approach, combining traditional instruction with VR-based simulation.

Feedback and reflection were facilitated through cueing strategies, guided questioning, and structured debriefing sessions using the PEARLS framework. The simulation design incorporated the five sequential phases of the 5E model—Engagement, Exploration, Explanation, Elaboration, and Evaluation. Engagement was promoted through active participation; exploration activities were based on realistic trauma scenarios; explanation involved contextual cues provided via interactive pop-up prompts; elaboration was supported through co-developed scenarios with nursing faculty and trauma specialists to ensure realism; and evaluation consisted of structured knowledge assessments, individual feedback, and guided debriefing sessions focusing on clinical reasoning and reflective practice.

#### 2.4.2. Development of the Program VR-Based Trauma Nursing Education Program for Clinical Nurses

The development and evaluation of the virtual reality (VR)-based trauma nursing education program in this study were conducted following the stages of analysis, design, development, implementation, and evaluation, as outlined in the ADDIE instructional design model. In the analysis stage, the learning needs of clinical nurses caring for trauma patients were identified, and a comprehensive literature review was conducted to draft the initial program content. In the design stage, learning objectives were established to strengthen nursing competencies in managing common trauma cases. Based on these objectives, essential nursing procedures were prioritized and incorporated into the content, which was organized into trauma-specific scenarios. Each scenario consisted of preliminary learning, a pre-quiz, VR simulation, and a post-quiz. The instructional strategy was guided by the 5E Learning Cycle model, allowing for immediate feedback and blended delivery through both traditional teaching and VR-based simulation. In the development stage, learning materials and scenarios were created and validated for content appropriateness by two adult nursing professors and five emergency nurses with more than five years of clinical experience. A pilot test was then conducted with five clinical nurses, and their feedback was incorporated to refine the final program materials. In the implementation stage, the program was delivered to the experimental group over four weeks, consisting of a two-hour VR-based trauma nursing education session (Week 1), a one-hour orientation and scenario discussion (Week 2), a two-hour core nursing skills training session related to the VR scenarios (Week 3), and a one-hour VR simulator operation session followed by a one-hour debriefing session (Week 4). Each simulation session followed the 5E structure of pre-quiz, simulation, and post-quiz, with participants required to achieve at least 80% on the quizzes, supported by immediate feedback and guided cues. Finally, in the evaluation stage, the effectiveness of the program was assessed using validated measures of knowledge, confidence, and emergency nursing competency, and structured debriefing sessions were conducted to promote reflection, clinical reasoning, and practical application of learning outcomes.

### 2.5. Data Collection

This study was conducted following approval from the Institutional Review Board (IRB) of the affiliated institution (IRB No. 40525-202112-HR-091-01), ensuring that all ethical considerations related to human participants were appropriately addressed. To maintain confidentiality, all research data were anonymized, and participants’ identities were protected throughout the study.

Data collection was carried out from 4 January to 24 March 2023, involving 21 clinical nurses in the experimental group and 21 in the control group. Prior to participation, the study was explained to potential participants by trained nurses, and those who voluntarily consented were sequentially assigned to either the experimental or control group. To minimize bias, participants were not informed of their group allocation.

### 2.6. Data Analysis

Data were analyzed using SPSS for Windows, version 24.0 (IBM Corp., Armonk, NY, USA). Descriptive statistics were used to summarize participants’ general characteristics, with frequencies and percentages reported for categorical variables and means with standard deviations for continuous variables. Homogeneity between the experimental and control groups in general characteristics was examined using the chi-square test or Fisher’s exact test, as appropriate. Pre-intervention homogeneity of the dependent variables was verified using independent samples *t*-tests. The effectiveness of the intervention was evaluated using repeated-measures analysis of variance (repeated-measures ANOVA) to assess changes in the dependent variables over time and between groups.

## 3. Results

### 3.1. General Characteristics of the Participants

The homogeneity test indicated no significant differences between the experimental and control groups in terms of age, clinical experience, and preferred educational methods (Table 1). The mean age was 32.3 years in the experimental group and 34.0 years in the control group. With respect to clinical experience, the majority of participants had more than five years of experience (88.2% in the experimental group and 82.4% in the control group). Regarding preferred educational methods, lectures were the most frequently selected in both groups, followed by practical training, group discussions, and question-and-answer sessions.

### 3.2. Homogeneity of Dependent Variables at Baseline

Independent samples *t*-tests confirmed baseline equivalence between the experimental and control groups. No significant differences were found in confidence in performing trauma care (t(32) = 0.61, *p* = 0.547, Cohen’s d = 0.21), knowledge of nursing for trauma (t(32) = −0.25, *p* = 0.798, Cohen’s d = 0.09), or trauma nursing competency (t(32) = −0.46, *p* = 0.644, Cohen’s d = 0.16), indicating homogeneity of the dependent variables at baseline (Table 2).

### 3.3. Effects of the Virtual Reality-Based Trauma Nursing Education Program

The results of the intervention are summarized in Table 3. The experimental group demonstrated greater improvements in trauma-related knowledge and confidence than the control group, whereas both groups improved similarly over time in emergency nursing competency. The experimental group demonstrated significant improvements across all outcome variables compared with the control group. Confidence scores increased from 52.00 ± 13.82 at baseline to 73.94 ± 10.64 after the intervention, whereas the control group improved from 48.70 ± 17.00 to 56.00 ± 15.78. Knowledge scores in the experimental group rose from 12.23 ± 2.51 to 16.29 ± 1.49, while the control group improved from 12.47 ± 2.78 to 14.23 ± 1.95. Competency scores increased from 110.76 ± 15.88 to 123.17 ± 19.00 in the experimental group and from 113.47 ± 17.85 to 123.82 ± 21.22 in the control group.

Repeated-measures ANOVA indicated significant main effects of time for knowledge (F = 79.52, *p* < 0.001), confidence (F = 73.18, *p* < 0.001), and competency (F = 17.74, *p* < 0.001), confirming improvements in both groups after the intervention. Moreover, significant interaction effects between group and time were observed for knowledge (F = 12.34, *p* = 0.001) and confidence (F = 18.59, *p* = 0.001), indicating greater gains in the experimental group. By contrast, the group-by-time effect for competency was not significant (F = 0.14, *p* = 0.706), indicating no between-group difference in the degree of change over time.

## 4. Discussion

This study developed and evaluated a virtual reality (VR)-based trauma nursing education program grounded in Jeffries’ simulation model and the 5E Learning Cycle. The program was designed to provide foundational evidence for the integration of VR training into trauma nursing education. The results demonstrated significant improvements in trauma-related knowledge and confidence among participants in the experimental group compared with those who received lecture-based education.

These findings are consistent with prior research reporting improved cognitive outcomes following simulation-based education. Similar benefits have been observed in cardiopulmonary emergency training for novice nurses [29], home-visit VR simulations for nursing students [30], and meta-analyses of VR-based nursing education [31]. Unlike many earlier studies that focused only on post-simulation knowledge acquisition, this study contributes by directly comparing the effectiveness of VR simulation with conventional instruction. This aligns with broader evidence that well-designed digital interventions can complement or replace traditional face-to-face education [32].

The enhancement in trauma-related knowledge may be attributed to the structured application of the 5E Learning Cycle, which promoted active engagement and problem-solving across diverse trauma scenarios. Immediate feedback, structured debriefing, and interactive prompts likely facilitated knowledge construction and clinical reasoning, consistent with the principles of the 5E model [28].

Confidence in trauma care also improved significantly in the experimental group. While some meta-analyses have reported limited effects of VR on self-efficacy [33], the blended design of this program—combining VR practice with instructor guidance, feedback, and debriefing—may explain the positive outcomes. By integrating exploration with structured explanation, the program appears to have reduced learning barriers and strengthened confidence.

In contrast, improvements in emergency nursing competency were observed in both groups, without significant group differences. Competency integrates knowledge, skills, and attitudes and typically develops through systematic, long-term education [34,35]. The absence of group differences may reflect confounding factors such as workplace experiences or concurrent training during the study period. It is also possible that the majority of participants, who had more than five years of experience, already possessed relatively high baseline competency. Longer interventions with novice nurses and larger samples may be needed to detect meaningful effects on competency.

Beyond the statistical results, this program holds important educational implications. VR simulation offers flexible, repeatable practice opportunities that are not constrained by clinical availability, allowing nurses to engage in independent learning and skill reinforcement. For educators, the integration of Jeffries’ model and the 5E Learning Cycle provides a structured framework for designing immersive learning environments that combine theoretical grounding with experiential practice. These features suggest that VR-based training can complement traditional education by reducing resource limitations, enhancing learner engagement, and ultimately contributing to safer and more effective trauma care.

This study has several limitations. The quasi-experimental design without randomization introduces potential selection bias. The sample size was small and drawn from three hospitals in a single metropolitan area, limiting generalizability. In addition, outcomes were assessed using self-report questionnaires, which may not fully capture actual clinical performance. These limitations should be considered when interpreting the findings.

## 5. Conclusions

This study systematically developed a VR-based trauma nursing education program using Jeffries’ simulation model and the 5E Learning Cycle. The program significantly improved trauma-related knowledge and confidence among clinical nurses, whereas its effect on competency was limited to improvements over time in both groups without significant between-group differences.

Despite these limitations, the study provides meaningful insights for nursing education. By integrating a theoretical framework with immersive VR simulation, the program demonstrates the feasibility of delivering flexible, repeatable, and engaging learning opportunities beyond traditional classroom or clinical settings. Such approaches may help overcome constraints in trauma education, particularly where access to diverse patient cases or resources is limited.

Nevertheless, the findings should be interpreted with caution. The quasi-experimental design, small sample size, and reliance on self-report measures limit generalizability and causal inference. Future randomized controlled studies with larger, more diverse populations and objective performance assessments are needed to validate the program’s impact and clarify its effectiveness in enhancing competency.

In summary, VR-based trauma nursing education appears to be a promising adjunct to conventional training, with demonstrated benefits for knowledge and confidence. With further refinement and rigorous testing, such programs may contribute to advancing the quality of trauma care and strengthening nurses’ preparedness for complex clinical situations.

## Figures and Tables

**Table 1:** General characteristics of the participants (*n* = 34).

Characteristics	Categories	Experimental (*n* = 17) *n* (%) or Mean ± SD	Control (*n* = 17) *N* (%) or Mean ± SD	x^2^ or F	*p*
Age		32.3 ± 5.1	34.05 ± 4.6	16.02	0.175
Clinical Experience	<1 Year	2 (11.8)	0(0)	4.54	0.305
	<2 Year	0 (0)	1 (5.8)		
	<3 Year	0 (0)	2 (11.8)		
	<5 Year	10 (58.8)	8 (47.1)		
	<10 Year	5 (29.4)	6 (35.3)		
Sex	-	-	-	-	-
Preferred Education	lecture	11 (64.8)	8 (47.1)	3.99	0.295
	discussion	2 (11.8)	1 (5.9)		
	Question-and-Answer	1 (5.9)	0 (0)		
	Performane	3 (17.6)	8 (47.1)		

**Table 2:** Pretest homogeneity between the experimental and control groups (*n* = 34).

Variable	Exp. (*n* = 17) Mean ± SD	Cont. (*n* = 17) Mean ± SD	t (df)	*p*	Cohen’s d	t
Knowledge of nursing for trauma patients	12.23 ± 2.51	12.47 ± 2.78	−0.25 (32)	0.798	0.21	
Confidence in performing trauma care	52.00 ± 13.82	48.70 ± 17.00	0.61 (32)	0.547	0.09	
Emergency nursing competency	110.76 ± 15.88	113.47 ± 17.85	−0.46 (32)	0.644	0.16	

**Table 3:** Pretest–post-test mean differences in knowledge, confidence, and competency in nursing care for trauma patients between the experimental and control groups over time (*n* = 34).

Characteristics	Exp. (*n* = 17) Mean ± SD	Cont. (*n* = 17) Mean ± SD	Source	x^2^ or F	*p*
Knowledge of nursing for patients with trauma					
pretest	12.23 ± 2.51	12.47 ± 2.78	Group	1.71	0.200
post-test	16.29 ± 1.49	14.23 ± 1.95	Time	79.52	<0.001
			Group × Time	12.34	0.001
Confidence in performing trauma care					
pretest	52.00 ± 13.82	48.7 ± 17.00	Group	5.12	0.031
post-test	73.94 ± 10.64	56.00 ± 15.78	Time	73.18	<0.001
			Group × Time	18.59	0.000
Emergency nursing competency					
pretest	110.76 ± 15.88	113.47 ± 17.85	Group	0.08	0.774
post-test	123.17 ± 19.00	123.82 ± 21.22	Time	17.74	<0.001
			Group × Time	0.14	0.706

## Data Availability

The data that support the findings of this study are available from the corresponding author upon reasonable request due to privacy concerns.
